# Impact of Resistance Training on Body Composition and Physical Function in Individuals with Down Syndrome: A Meta-Analysis

**DOI:** 10.3390/diagnostics14232620

**Published:** 2024-11-21

**Authors:** Hou-Ting Kuo, Ting-Hsu Lee, Li-An Su, Li-Yun Wu, Ping-Yan Mao, Ciao-Ci Yang

**Affiliations:** 1 Department of General Medicine, China Medical University Hospital, Taichung 404, Taiwan; 2 Department of Medical Education, Taichung Veterans General Hospital, Taichung 407, Taiwan; timvm4326@gmail.com; 3 Department of Otorhinolaryngology-Head and Neck Surgery, China Medical University Hospital, Taichung 404, Taiwan; lilliansu1019@gmail.com; 4 Department of Pediatrics, Mackay Memorial Hospital, Taipei 104, Taiwan; catherine87216@gmail.com; 5 Department of Occupational Therapy, Chang Gung University, Taoyuan 333, Taiwan; j605404207@gmail.com; 6 Department of Chinese Medicine, China Medical University Hospital, Taichung 404, Taiwan

**Keywords:** body composition, energy expenditure, metabolic disorders, muscle tone, physical activity

## Abstract

**Objectives:** Down syndrome (DS) is associated with challenges like increased fat mass and reduced muscle tone. This study aims to analyze the impact of resistance training on improving muscle strength and physical function in individuals with DS. **Methods:** We conducted a comprehensive search of PubMed, Scopus, Google Scholar, Cochrane Library, and China National Knowledge Infrastructure for studies published up to August 2023. Standardized mean differences and 95% confidence intervals were used to evaluate the efficacy of resistance training. **Results:** Eight randomized controlled trials (RCTs), including 127 participants with DS, met the inclusion criteria. Resistance training significantly improved muscle strength in the knee extensors (SMD: 1.009, 95% CI: 0.505–1.513), knee flexors (SMD: 1.133, 95% CI: 0.106 to 2.16), and upper limbs (SMD: 0.748, 95% CI: 0.152–1.343). The SMD for upper limb function was 0.385 (95% CI: 0.004–0.765), showing a small improvement. For walking ability, the SMD was 0.259 (95% CI: −0.171–0.690), and for stair climbing ability, the SMD was 0.257 (95% CI: −0.189–0.703), both indicating no significant changes. Improvements in upper limb physical function were significant, but no notable improvements were seen in lower limb functions. **Conclusions:** Resistance training for more than four weeks enhances muscle strength in individuals with DS, with greater gains seen in younger participants. While improvements were noted in upper limb physical function, lower limb function remained largely unchanged. These findings suggest that resistance training is a valuable exercise for improving muscle strength and physical function in individuals with DS.

## 1. Introduction

Down syndrome (DS) results from an additional copy of chromosome 21 and, in turn, causes distinctive physical challenges and cognitive limitations [1]. Studies have shown that, in addition to intellectual shortcomings, individuals with DS often experience increased body weight and higher fat levels, and distinct muscle mass and fat distribution [2,3].

The body composition of patients with DS is complicated by physiological factors, including reduced muscle tone, lower leptin levels, systemic inflammation, metabolic disorders, and slow metabolism [4]. Furthermore, individuals with DS often have lower resting energy expenditure, providing a physiological basis for their predisposition to weight gain [5]. Concurrently, comorbidities and poor dietary habits contribute to weight gain among these patients [6]. Additionally, cognitive challenges may affect their awareness and ability to maintain healthy dietary habits and engage in moderate exercise, making them less likely to actively control their weight and participate in physical activity. This exacerbates the problem of excessive weight among patients with DS [7].

The effectiveness of exercise in improving functioning performance in individuals with DS is controversial, marked by inconsistent research findings [8,9,10]. Moreover, most studies analyzed the effects of combined exercise on individuals with DS, including aerobic exercise [8], resistance training [11], and aquatic exercises [12], giving less attention to the clinical significance of single types of exercise.

Resistance training is a simple, cost-effective, and convenient exercise method that requires less space and time, making it more accessible for most people. Moreover, we believe that it is also beneficial for patients with DS. Since compliance is a crucial factor among individuals with DS, understanding how resistance training affects the body composition of these patients is critical. Thus, this study aimed to analyze various studies to better understand the impact of resistance training on the body composition of individuals with DS. Although several studies have reported resistance training among individuals with DS [13,14,15], as well as various meta-analyses in healthy people [16,17,18], to the best of our knowledge, no meta-analysis focused on the clinical effect of resistance training on physical function and muscle strength in patients with DS. The current study emphasizes relevant indicators of progress, such as muscle strength, upper limb function, and the ability to walk and climb stairs. Our study can help physical and occupational therapists and rehabilitation physicians make a steadier and more effective plan to improve the health of individuals with DS. Moreover, this study presents new evidence that supports the benefits of resistance training for patients with DS.

## 2. Methods

Our study protocol was performed according to the Preferred Reporting Items for Systematic Reviews and Meta-analyses (PRISMA) statements [19].

### 2.1. Literature Search Strategy

**Figure 1:**
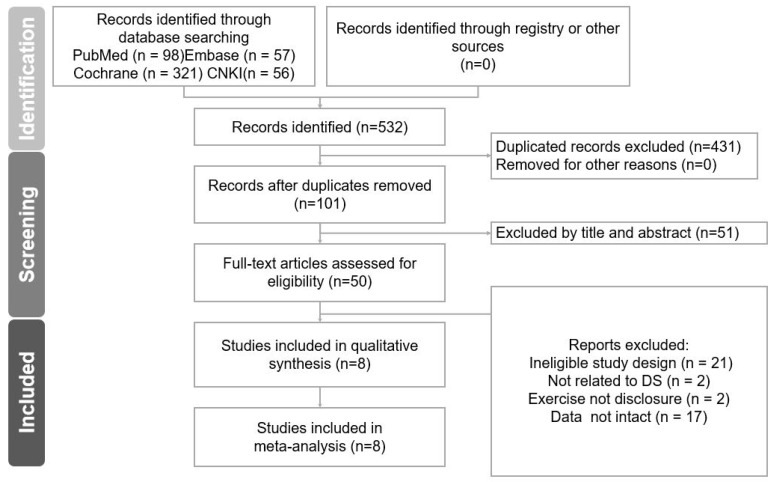
The process of study selection. Overall, 532 studies were initially identified through electronic searches across all databases. After removing duplicates, 101 articles remained. Of these, 51 studies were excluded because of their title or abstract. Additional exclusions were made for studies with ineligible designs, durations of less than 4 weeks, unclear resistance training methods, or incomplete BMI or body fat data. Ultimately, 8 articles, comprising 127 participants, met the inclusion and exclusion criteria and were included in this meta-analysis.

The databases PubMed, Scopus, Google Scholar, Cochrane Library, and China National Knowledge Infrastructure were searched using the following search terms: “Down syndrome”, “resistance training”, “strength training”, “weight training”, and “exercise”. Furthermore, a manual search for original studies that may have been missed by the database searches was conducted. Additionally, citations within the identified literature were examined. Only original articles published in peer-reviewed journals from the earliest record to August 2023 were included in our current meta-analysis. There was no exclusion based on language (Figure 1).

### 2.2. Eligibility Criteria

The following inclusion criteria were applied to determine if the included studies would be involved in our meta-analysis: studies with an enrollment of patients with DS studies that include a resistance training course of at least 4 weeks in duration and studies with quantitative measurement of muscle strength or body composition.

The following types of studies were excluded: non-RCT studies, studies in which full text could not be obtained or no data available, animal studies, and studies with patients who did not receive training for more than 4 weeks.

Our primary outcome was to compare muscle strength, physical performance, and body composition before and after resistance training. The standardized mean difference (SMD) was used to estimate the efficacy of training.

### 2.3. Study Selection

The title and abstracts of all the identified studies were reviewed independently by two researchers (H.T.K and C.C.Y). Subsequently, a comprehensive evaluation of the full text of all potentially relevant studies was conducted to determine if they met the criteria for inclusion or exclusion.

### 2.4. Data Collection and Risk of Bias Assessment

When the studies were selected and included, two independent researchers (H.T.K and C.C.Y) extracted specific data from each study. The data included the study design, first author, publication year and journal, study location, patient demographics (sex, age), follow-up duration, training progression, frequency of resistance training, and muscle strength during each follow-up. The risk of bias for each included study was assessed by the same two researchers according to the Cochrane risk of bias tool 2 and categorized as “low”, “unclear”, or “high”. A study was considered to have a high risk of bias if at least one domain was rated as high.

A preliminary selection of studies was conducted by two reviewers (H.T.K and C.C.Y) based on their relevance and adherence to the inclusion criteria. Discrepancies in study selection were resolved through discussion, and a study was deemed eligible if one of the reviewers considered it to meet the criteria. Subsequently, data extraction and qualitative assessment of the included studies were performed by the same two reviewers.

### 2.5. Data Synthesis and Analysis

Study findings were presented according to the PRISMA guidelines (Supplementary material File S1). For articles that provided data segmented into various treatment groups, each set of data was considered as distinct treatment categories during analysis. All statistical analyses were carried out using the Comprehensive Meta-analysis software version 3.0. Pooled estimates were computed along with a 95% confidence interval (CI). *p* < 0.05 indicated statistical significance. Heterogeneity was characterized as significant if the I^2^ value exceeded 50.0%. Prior to delving into quantitative analysis, a random-effects model was employed. The I^2^ statistic was utilized to quantify this heterogeneity. The variation may stem from factors such as varying training durations, follow-up durations, participant ages, and the study’s country of origin. Consequently, predefined subgroup analyses were conducted for patients based on these criteria. Additionally, funnel plots and Egger’s tests were used to evaluate the potential influence of publication bias.

## 3. Results

### 3.1. Study Search and Characteristics of Included Patients

Figure 1 presents the study selection process. Overall, 532 studies were identified from the electronic search of all databases. Then, 101 articles were examined after duplicates were excluded. Among these, 51 studies were excluded owing to their title or abstract. The other studies were excluded because of ineligible study design, duration < 4 weeks, resistance project method no disclosure, and data of BMI or body fat not intact. Finally, 8 articles (consisting of 127 patients) screened based on the aforementioned inclusion and exclusion criteria were included in this meta-analysis. Notably, the actual participants in the studies by Rosety-Rodriguez in 2021 and Diaz in 2021 are the same group. Therefore, when conducting statistical analyses, they will be treated as the same cohort. Table 1 shows all the characteristics of the included studies.

### 3.2. Quality Assessment and Risk of Bias

Risk of bias assessment [12] is displayed in Table 2. The overall risk of bias in the included studies was mixed. Two of the included studies were rated as having some concerns about deviations from the intended intervention. Hence, two studies were regarded as concerned with the overall risk of bias, and another showed low risk.

### 3.3. Outcomes

A pooled analysis of all eight studies was conducted to assess overall changes before and after resistance training. The primary outcomes included muscle strength of the knee flexors, knee extensors, and upper limbs. The secondary outcomes were the physical function of the upper and lower limbs, fat mass, and waist circumference. Lower limb function was further subdivided into walking ability and stair climbing ability. Table 1 summarizes the different results presented in various studies.

**Figure 2:**
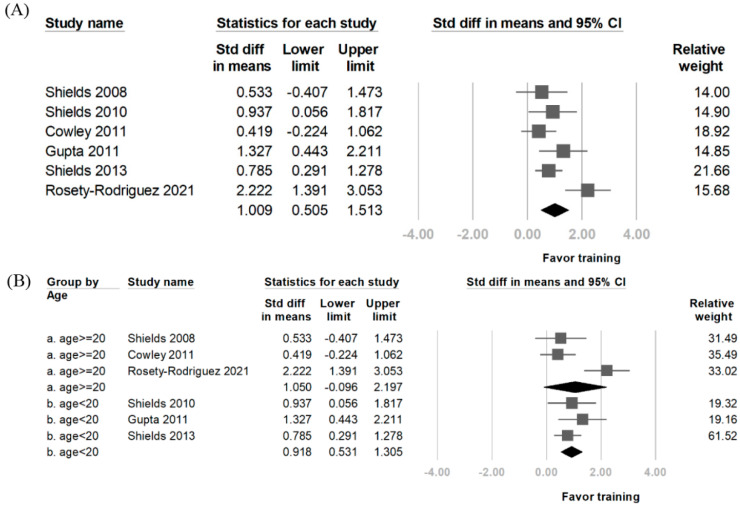
Forest plot of the effects of resistance training on quadriceps muscle strength. (**A**) Overall effect of resistance training on quadriceps strength across all studies. (**B**) Subgroup analysis based on different ages. If the tip or straight line in the diagram crosses zero, it indicates that the result is not statistically significant [9,11,15,20,21,23].

**Figure 3:**
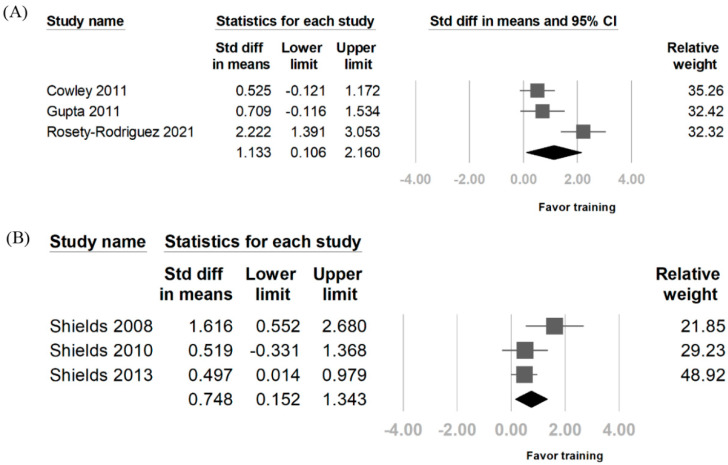
Forest plot of the effects of resistance training on knee flexor and upper limb muscle strength. (**A**) Analysis of the impact on knee flexor strength. (**B**) Analysis of the impact on upper limb major muscle strength. Pooled data demonstrate significant improvements in knee flexors and upper limb strength after resistance training [9,11,15,20,21,23].

The results of the included studies are shown in Table 3. The SMD post-treatment from training regarding knee extensor changes was 1.009 (95% CI: 0.505–1.513). Regarding heterogeneity of the included study, the I^2^ was 62% (Figure 2). To determine whether the age of patients with DS would be a source of confounding, a separate pooled analysis was performed. The subgroup analysis yielded an SMD of 1.050 (95%CI: −0.096–2.197) for individuals aged > 20 years. In those aged < 20 years, the SMD was 0.918 (95% CI: 0.531–1.305). Three articles were included in the analysis assessing changes in the knee flexors after training. The SMD was 1.133 (95% CI: 0.106–2.16), with an I^2^ of 81.56%. For the upper limb muscle strength, three articles were included, and the SMD was 0.748 (95% CI: 0.152–1.343), with an I^2^ of 45.16% (Figure 3).

**Figure 4:**
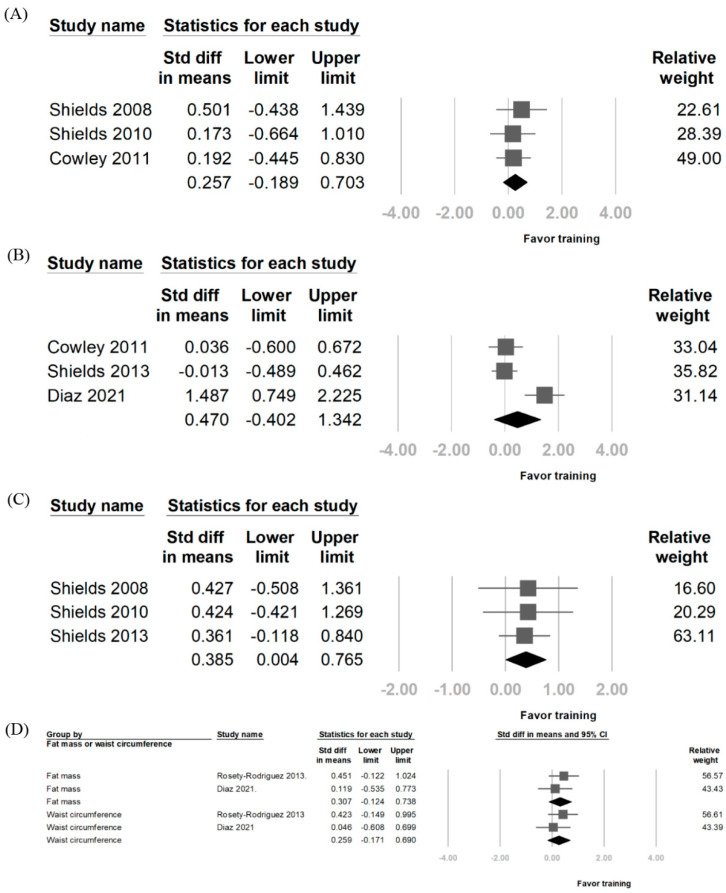
Forest plot of functional performance and body composition after resistance training. (**A**) Functional tests of stair climbing. (**B**) Functional tests of walking. (**C**) Functional tests of upper limbs. (**D**) Body composition outcomes, including fat mass and waist circumference [9,11,15,21,22,24].

Three articles were included for each activity for the physical functional analysis. The SMD for upper limb function was 0.385 (95% CI: 0.004–0.765), with an I^2^ of 0%. The SMD for walking ability was 0.259 (95% CI: −0.171–0.690), with an I^2^ of 0%. The SMD for stair climbing ability was 0.257 (95% CI: −0.189–0.703), with an I^2^ of 0% (Figure 4). Notably, the so-called “upper limb function”, as referenced in the three included studies, primarily refers to the speed of placing items onto shelves. This can be envisioned as lifting and organizing items onto a shelf.

Two articles were included for each measurement for the analysis of changes in fat mass and waist circumference after training. The SMD for fat mass was 0.307 (95% CI: −0.124–0.738), with an I^2^ of 0%. The SMD for waist circumference was 0.259 (95% CI: −0.171–0.690), with an I^2^ of 0% (Figure 4).

## 4. Discussion

To the best of our knowledge, this is the first meta-analysis to explore the clinical significance of resistance training on muscle strength, body fat levels, and physical function in individuals with DS.

### 4.1. Novel Findings

This study found that resistance training for a minimum of 6 weeks can enhance muscle strength of the upper and lower limbs of individuals with DS. However, subgroup analysis revealed that significant improvement in lower limb extensor strength occurred in patients aged < 20 years but not in those aged > 20 years. Additionally, the analysis showed no significant changes in fat mass or waist circumference following resistance training. While resistance training improved the physical function of the upper limbs, it did not show significant improvements in lower limb functions, such as walking and stair climbing.

### 4.2. Clinical Implications

Muscle strength is significantly linked to future health outcomes [25]. Individuals with intellectual disabilities (ID) often exhibit lower muscle strength [26], and research has found that the areas of the brain responsible for motor function are affected in individuals with intellectual disabilities [27]. This may be because individuals with ID are less active than the general population [28], or awareness regarding the connection between exercise and health is lacking. Individuals with DS face intellectual challenges and experience hormonal imbalances and metabolic disorders that lead to higher fat and lower muscle mass [29], impacting their health and often causing functional limitations because of muscle weakness [30]. Taylor et al. indicated that progressive resistance training was feasible and safe for people with DS with excellent adherence and minimal adverse effects [31]. Our study demonstrates that resistance training can effectively enhance muscle strength in the upper and lower limbs of individuals with DS. This result offers compelling evidence to patients with DS and their caregivers, who may be hesitant or believe exercise is unsuitable [32], to encourage strength training for individuals with DS.

Additionally, our subgroup analysis revealed that age affects training outcomes. Individuals with DS aged < 20 years showed significant improvements after strength training, whereas those aged > 20 years did not. This may be related to the study by Cioni et al., which found that by age 14 years, adolescents with DS do not demonstrate muscle strength increases that typically occur by this age. The study indicated that this could be linked to hormone-mediated factors or the premature aging seen in individuals with DS [33,34]. Additionally, some studies have shown that age is negatively associated with improvements after resistance training [35], indicating that the premature aging experienced by individuals with DS may significantly impact their progress in resistance training. This emphasizes the importance of early intervention in exercise for individuals with DS.

Structural differences in individuals with DS, such as a short neck, small hands and feet, and reduced height, often result in impaired motor learning at a young age [36]. This can negatively impact motor functioning and subsequently decrease independence in adulthood. The current study showed significant improvement in upper limb function following training, although no significant improvement was noted in lower limb functions such as walking and stair climbing. This lack of improvement may be because the walking and stair-climbing disability of individuals with DS is influenced by muscle strength and coordination, poor cardiovascular fitness, intellectual disability, hypotonia, and balance [37,38]. The diversity among individuals makes individualized and more comprehensive exercise programs more effective than a standardized or “one size fits all” model. A systemic review revealed that aerobic exercise showed significant improvements in dynamic balance, whereas combined exercise, which included simultaneous aerobic and resistance training, significantly increased dynamic and static balance in adults with DS [39].

Another analysis from this study showed that among the three studies on walking ability, only Cowley [9] et al. study did not involve carrying weights. When Cowley et al. [9] study was excluded, and a subgroup analysis was conducted, the results showed significant improvement (SMD was 0.426; 95% CI: 0.027–0.826), indicating that while strength training alone may not enhance walking ability, it can improve the ability to walk with loads. Physical function is associated with activities of daily living, such as washing dishes, showering, and using the restroom [40]. According to the results of this study, resistance training can enhance the ability of individuals with DS to care for themselves. Regarding social participation, individuals with DS are more often employed in physically demanding jobs than in jobs that involve cognitive effort. Thus, increased muscle strength and physical function may significantly benefit their work.

Lastly, no significant changes were observed in body fat and waist circumference, which is consistent with the results of other studies showing that aerobic exercise has a more pronounced effect on fat reduction than strength training [41,42]. Our previous research found that swimming training for 8–36 weeks significantly reduced body fat percentage in individuals with DS [43].

In summary, the present study provides guidance on exercise choices for individuals with DS. Strength training is an accessible and effective option for increasing muscle strength and improving physical function. However, for fat reduction, other training modalities, such as aerobic exercise, may prove to be more effective than strength training.

### 4.3. Comparisons to Other Studies

The present meta-analysis included 8 RCTs, with a total of 127 patients with DS. Several studies have discussed whether exercise is beneficial for individuals with DS. Our findings are consistent with those of a 2023 meta-analysis [39], which showed that combined exercise significantly increased muscle strength in the upper and lower limbs. The difference is that this study exclusively examined resistance training, whereas the other meta-analysis included combined exercise, which incorporated both aerobic and resistance training. Moreover, Gonzalez et al. found potential benefits of resistance training on upper and lower limb muscle strength [44]. Our study further explores the impact of age on training outcomes and delves deeper into the effects of resistance training on the physical function of the upper and lower limbs.

As regards the impact of exercise on body fat, a meta-analysis conducted in 2022 found that resistance training significantly reduced body fat in healthy adults [45]. Further, Salsa et al. discovered that combined training did not reduce body fat in people with intellectual disabilities [46]. Our previous study found that swimming significantly reduced body fat in individuals with DS [42]. To our knowledge, no meta-analysis has explored the effects of resistance training on body fat and waist circumference, specifically in individuals with DS.

### 4.4. Strength and Limitations

This meta-analysis had several strengths. It provides a comprehensive pooled analysis of the clinical effects of resistance training on individuals with DS, identifying improvements in muscle strength and examining the impact of age on these gains. More importantly, this study assessed whether exercise could enhance physical function, a critical factor for individuals with DS, and specifically analyzed the effects of resistance training on the physical functions of the upper and lower limbs. Additionally, the findings indicate that resistance training alone may not significantly impact body fat and waist circumference. These insights can inform exercise choices for individuals with DS pursuing different goals and bolster caregivers’ confidence in promoting resistance training.

Nevertheless, this study had some limitations. One limitation is the limited number of studies included in our meta-analysis, which comprised only eight RCTs. However, it should be noted that the scarcity of studies on this topic limited our selection, as more research focused on combined exercise rather than resistance training alone. Additionally, among the three studies assessing walking ability, one did not involve weight-bearing, whereas the other two did. Weight-bearing walking may be influenced by upper limb strength, potentially affecting the accuracy of lower limb assessments. Considering this, a subgroup analysis excluding the non-weight-bearing study was conducted, and it was found that the results changed from nonsignificant to significant improvement. However, this statistical result should be interpreted with caution, as it only includes two studies. More comprehensive analyses will be possible as further research in this area becomes available.

Future research could explore the effects of resistance training on a broader range of outcomes, such as balance, cognitive function, coordination, and cardiovascular function. These factors may influence the physical functions of individuals with DS, such as walking and performing activities of daily living, which in turn could significantly impact their quality of life and social participation.

## 5. Conclusions

Our meta-analysis found that resistance training for more than 4 weeks leads to significant improvements in upper and lower limb muscle strength in individuals with DS, with age also being a factor influencing strength outcomes. Although resistance training was beneficial for upper limb physical function, it did not have a significant impact on lower limb function. Additionally, resistance training did not significantly affect fat mass or waist circumference in individuals with DS. These findings provide healthcare professionals and caregivers greater confidence in encouraging resistance training in individuals with DS. Future research could explore additional outcomes related to the impact of resistance training on physical function.

## Figures and Tables

**Table 1:** Characteristics of included studies.

Study	Country	Population	Study Design	Duration	Patients	Mean Age	Training Program	Outcome Measurements
Shields, 2008 [11]	Australia	Adult	RCT	10 weeks	9	25.8	Weight machines performed twice a week for 10 weeks	– 1 RM: Chest press and leg press – Muscular resistance: repetitions of chest and leg press with 50% of 1 RMTimed up and down stairs test – Grocery shelving task
Shields, 2010 [15]	Australia	Adolescents	RCT	10 weeks	11	15.9	Weight machines performed twice a week for 10 weeks	– 1 RM: Chest press and leg press – Timed up and down stairs test – Grocery shelving task
Cowley, 2011 [9]	USA	Adult	RCT	10 weeks		29	Weight machines performed twice a week for 10 weeks	– Knee extensor and flexor isometric and isokinetic strength – Functional tasks of daily living: Chair rise, stair ascent, walk speed – Peak aerobic capacity
Gupta, 2011 [20]	India	Adolescents	RCT	6 weeks	12	13.5	Weight machines performed three times a week for 6 weeks (only lower limb muscle included)	– Lower limb strength – Balance
Shields, 2013 [21]	Australia	Adolescents	RCT	10 weeks	34	17.7	Weight machines performed twice a week for 10 weeks	– 1 RM: Chest press and leg press – Weighted box stacking test – Weighted pail carry test
Rosety-Rodriguez, 2013 [22]	Spain	Adult	RCT	12 weeks	24	23.7	Weight machines performed three times a week for 12 weeks	– Fat-free mass percentage – Waist circumference – Timed get-up-and-go (TGUG) test
Rosety-Rodriguez, 2021 ^a^ [23]	Spain	Adult	RCT	12 weeks	18	28.4	Weight machines performed three times a week for 12 weeks	– Peak torques of knee extensors and flexors – Blood antioxidants – Handgrip strength
Diaz, 2021 ^a^ [24]	Spain	Adult	RCT	12 weeks	18	28.4	Weight machines performed three times a week for 12 weeks	– Blood test Body composition: Fat mass, muscle mass – Nutritional Intake Record – Weighted pail-carry test

^a^ Same study group in two papers.

**Table 2:** Risk of bias summary for each study based on the Cochrane Bias Assessment Tool.

Risk of Bias Assessment	Shields, 2008 [11]	Shields, 2010 [15]	Cowley, 2011 [9]	Gupta, 2011 [20]	Shields, 2013 [21]	Rosety-Rodriguez, 2013 [22]	Rosety-Rodriguez, 2021 [23]	Diaz, 2021 [24]
Randomization process	Low risk	Low risk	Low risk	Low risk	Low risk	Low risk	Low risk	Low risk
Deviations from intended intervention	Some concerned	Low risk	Low risk	Low risk	Low risk	Low risk	Low risk	Low risk
Missing outcome data	Low risk	Low risk	Low risk	Low risk	Low risk	Low risk	Low risk	Low risk
Measurement of the outcome	Low risk	Low risk	Low risk	Low risk	Low risk	Low risk	Low risk	Low risk
Selection of the reported result	Low risk	Low risk	Low risk	Low risk	Low risk	Low risk	Low risk	Low risk
Overall risk of bias	Some concerned	Low risk	Low risk	Low risk	Low risk	Low risk	Low risk	Low risk

**Table 3:** Analysis and Subgroup analysis of standard means differences in different outcomes. Italic is used to indicate the classification of muscles or functions in different locations.

Subgroup	Standard Mean Difference	95% Confidence Interval
*Lower limbs muscle strength*		
Knee extensors	1.009	0.505 to 1.513 *
Age ≥ 20	1.050	−0.096 to 2.197
Age < 20	0.918	0.531 to 1.305 *
Knee flexors	1.133	0.106 to 2.16 *
*Upper limbs muscle strength*	0.748	0.152 to 1.343 *
*Lower limb’s physical function*		
Stair climbing	0.257	−0.189 to 0.703
Walking ability	0.259	−0.171 to 0.690
*Upper limb’s physical function*	0.385	0.004 to 0.765 *
*Body composition*		
Fat mass	0.307	−0.124 to 0.738
Waist circumference	0.259	−0.171 to 0.690

* indicates a significant difference.

## Data Availability

The data generated during and analyzed in this article are available from the corresponding author without undue reservation.

## Supplementary Materials

The following supporting information can be downloaded at: https://www.mdpi.com/article/10.3390/diagnostics14232620/s1, File S1: PRISMA 2020 Check list.
